# Tumor cell MT1-MMP is dispensable for osteosarcoma tumor growth, bone degradation and lung metastasis

**DOI:** 10.1038/s41598-020-75995-6

**Published:** 2020-11-05

**Authors:** Signe Z. Ingvarsen, Henrik Gårdsvoll, Sander van Putten, Kirstine S. Nørregaard, Oliver Krigslund, Josephine A. Meilstrup, Collin Tran, Henrik J. Jürgensen, Maria C. Melander, Carsten H. Nielsen, Andreas Kjaer, Thomas H. Bugge, Lars H. Engelholm, Niels Behrendt

**Affiliations:** 1 Finsen Laboratory, Rigshospitalet/Biotech Research and Innovation Center (BRIC), University of Copenhagen (UCPH), Ole Maaløes Vej 5, 2200 Copenhagen N, Denmark; 2 BRIC, UCPH, Copenhagen, Denmark; 3 Proteases and Tissue Remodeling Section, National Institute of Dental and Craniofacial Research, National Institutes of Health, Bethesda, MD USA; 4 In Vivo Pharmacology, Symphogen A/S, Ballerup, Denmark; 5 Department of Clinical Physiology, Nuclear Medicine & PET and Cluster for Molecular Imaging, Rigshospitalet and UCPH, Copenhagen, Denmark

**Keywords:** Cancer, Molecular biology

## Abstract

The membrane-anchored matrix metalloprotease MT1-MMP is a potent collagenolytic enzyme with a well-established role in extracellular matrix turnover and cellular invasion into collagen-rich tissues. MT1-MMP is highly expressed in various types of cancer and has been demonstrated to be directly involved in several stages of tumor progression, including primary tumor growth, angiogenesis, invasion and metastasis. Osteosarcoma is the most common type of primary bone cancer. This disease is characterized by invasive tumor growth, leading to extensive bone destruction, and metastasis to the lungs. The tumor cells in human osteosarcoma display a strong expression of MT1-MMP, but the role of MT1-MMP in osteosarcoma progression is currently unknown. In this study, we investigated the role of MT1-MMP during various stages of osteosarcoma development. We utilized an optimized orthotopic murine osteosarcoma model and human osteosarcoma cells in which the MT1-MMP gene was knocked out using CRISPR/Cas9. We observed a strong expression of MT1-MMP in wildtype cells of both primary tumors and lung metastases, but, surprisingly, MT1-MMP deficiency did not affect primary tumor growth, bone degradation or the formation and growth of lung metastases. We therefore propose that, unlike findings reported in other cancers, tumor-expressed MT1-MMP is dispensable for all stages of osteosarcoma progression.

## Introduction

Processes of tissue destruction and degradation of the extracellular matrix (ECM), associated with cancer invasion and metastasis, are critically dependent on pericellular and extracellular proteolytic activity. Many of the proteases involved in these processes belong to the matrix metalloprotease (MMP) group. A collagenolytic MMP which has received particular attention in this respect is the membrane-associated MT1-MMP (MMP-14), which is upregulated in several cancers^1^. MT1-MMP efficiently facilitates the turnover of extracellular matrices by directly degrading collagens and other ECM substrates^2,3^, as well as by initiating a cascade mechanism involving the activation of pro-MMP-2 and pro-MMP-13^4–6^. Therefore, it is not surprising that MT1-MMP has been found to be involved in invasion and metastasis in several cancers, e.g. in studies on mouse cancer models^7–10^.

In cancers of the carcinoma type, a complicated tumor-stroma interplay governs the expression of MT1-MMP on tumor cells, stromal cells, or both, with substantial variation among cancer types and a pronounced variation within each given type^11^. However, studies in mouse tumor models suggest that, e.g. in mammary carcinomas, it is the tumor cell expression of MT1-MMP that is particularly decisive for invasion and metastasis^10,12^.

Sarcomas have been studied less intensively than carcinomas with respect to the in vivo function of specific proteases, including MT1-MMP, probably due to their lower incidence in humans^13^. However, possibly due to their mesenchymal lineage, most sarcomas display a strong expression of MT1-MMP on the tumor cells. Indeed, sarcomas have the highest expression of this protease of all cancers as determined by mRNA quantification, with osteosarcomas and chondrosarcomas presenting with the highest expression within the sarcoma group^13^.

Based on the importance of tumor cell-expressed MT1-MMP in carcinomas, a similar critical function would appear likely in sarcomas. Osteosarcoma is the most common type of primary bone cancer and the consistent expression of MT1-MMP on the tumor-cells of osteosarcoma has been confirmed by immunohistochemistry^14^. Due to the strong metastatic capacity, the poor prognosis and the current lack of efficient treatment options, there is a strong need for development of novel therapies against osteosarcoma^15^. This dictates a need for additional knowledge on the mechanisms of invasion and metastasis associated with this disease. In this paper, we have focused on the role of MT1-MMP on osteosarcoma cells in vivo, using an established mouse model with human tumor cells displaying a high expression of the protease. Surprisingly, we find that MT1-MMP is dispensable for tumor growth, invasive bone destruction and experimental metastasis, with no difference observed between MT1-MMP-positive tumors and tumors manipulated to be deficient for the protease.

## Results

### Development of an optimized 143B osteosarcoma model

**Figure 1:**
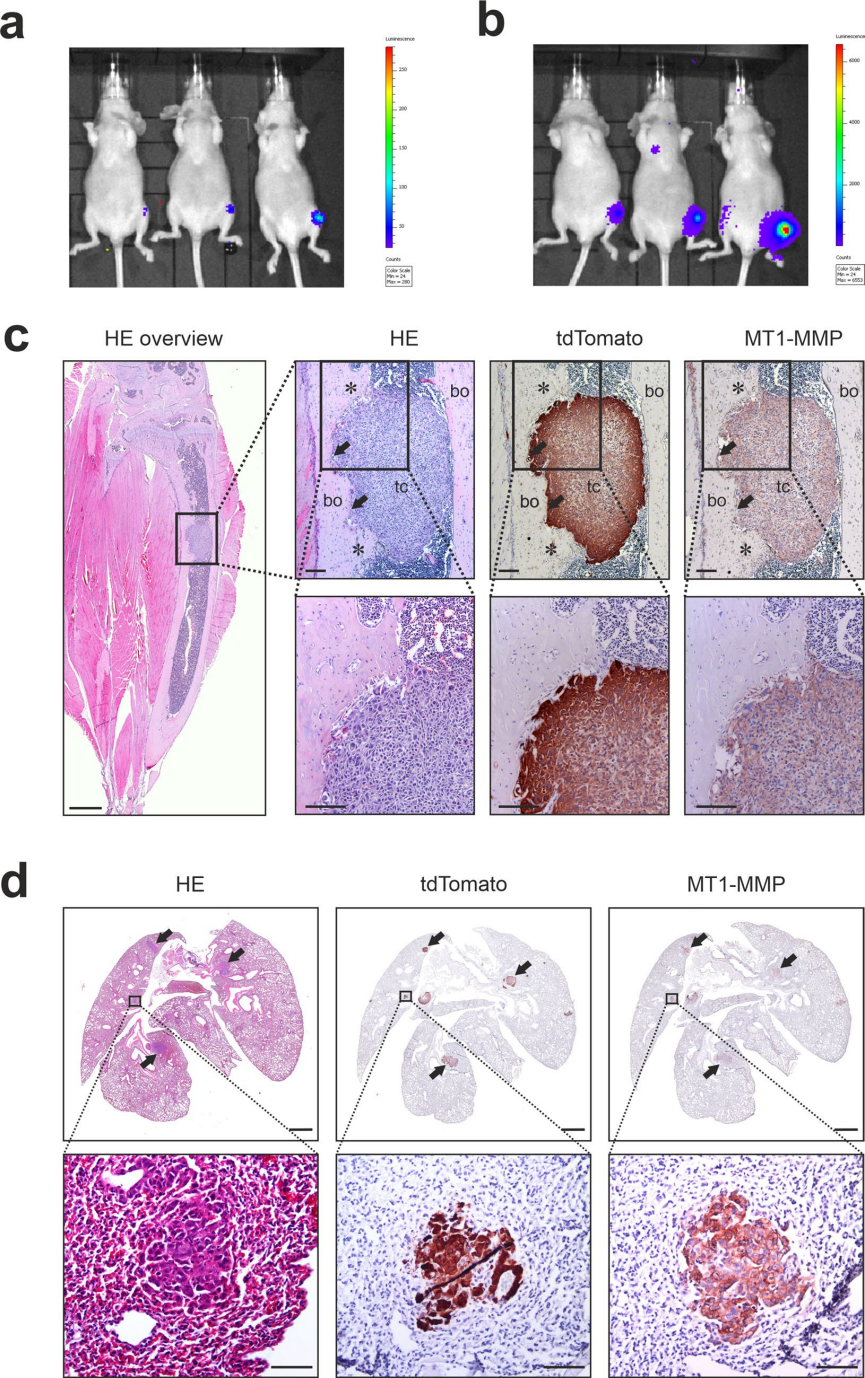
Expression of MT1-MMP in a bioluminescent 143B osteosarcoma model. (**a**,**b**) Detection of primary tumors and lung metastasis by non-invasive bioluminescent imaging in vivo. Each mouse was injected with 3.3 × 10^4^ 143B-luc2/tom cells into the left tibia. (**a**) Examples showing primary tumor signals 1 day after injection. (**b**) The same mice as shown in (**a**), examined 7 days after injection. A metastasis signal is noted in the center mouse. (**c**) Histological characterization of a primary tumor derived as in (**a**). Left: a section of a tibia with a tumor stained with haematoxylin and eosin (HE) is shown in low magnification (bar = 1 mm). Right: sections of the primary tumor stained with HE or immunostained for tdTomato and MT1-MMP. The sections are shown in two different magnifications; the box in the upper row indicates the region shown at higher magnification below (bars = 100 µm). The tdTomato immunostain clearly distinguishes tumor cells from the surrounding bone and bone marrow. The MT1-MMP immunostain reveals a pronounced expression of MT1-MMP in the tumor cells. Areas of bone degradation (arrow) and new bone formation (asterisk) are indicated; bo = bone; tc = tumor cells. For verification of the specificity of MT1-MMP immunostaining, see Fig. 4. (**d**) Histological characterization of 143B lung metastases resulting from intra-tibial injection of 143B-luc2/tom cells. Sections stained with HE or immunostained for tdTomato and MT1-MMP are shown as low magnification overviews (upper; bars = 1 mm) and higher magnification of the indicated region (lower; bars = 50 µm). Overview pictures are whole slide scans of stained sections, while high magnification photographs were taken directly through the microscope. Examples of metastasis nodules are indicated in the overview pictures (arrows). Even small 143B metastasis nodules could easily be detected by tdTomato immunostain and MT1-MMP was strongly expressed by the metastatic tumor cells. For specificity of MT1-MMP immunostaining, see Fig. 5.

To investigate the role of MT1-MMP in osteosarcoma disease development, we based our study on a murine orthotopic osteosarcoma model in which human 143B osteosarcoma cells are injected into the tibia of mice (Fig. 1). The 143B model is a widely used osteosarcoma model and is characterized by high tumor take, bone degradation and metastasis to the lungs^16–18^. To enable the study of both early primary tumor growth, bone degradation and lung metastasis, we established conditions where injection of a low number of tumor cells into the tibia allowed us to follow primary tumor growth and metastasis over time in vivo. For this purpose, we generated a 143B cell line with stable expression of luciferase (as well as tdTomato for subsequent analyses) and used bioluminescent imaging in vivo for non-invasive detection and quantification of tumor growth. By this approach we could detect tumor cells within the tibia less than 24 hs after injection of a low number of cells. In pilot experiments with varying cell numbers and a small injection volume, we found that a high tumor take (> 80%) with well-defined intra-tibial tumors was obtained after injection of 3.3 × 10^4^ cells (Fig. 1a; see also “Methods”). The growth of these tumors could be quantitatively recorded over time (see later and Fig. 3). In some mice, lung metastases could also be detected by bioluminescent imaging (Fig. 1b).

### MT1-MMP is expressed by 143B orthotopic tumors and lung metastases

Human osteosarcomas are characterized by both bone destruction and production of new bone matrix^19^. A histological characterization of the primary 143B tumors showed evident areas of both bone degradation and new bone formation (example shown in Fig. 1c). Since the tumor cells express tdTomato, tdTomato immunohistochemistry (IHC) could be used to distinguish tumor cells from the surrounding bone and host cells (Fig. 1c). Reassuringly, when staining the same specimens with antibody against MT1-MMP, the 143B tumors displayed a clear expression of MT1-MMP (Fig. 1c), very similar to the pattern found in human osteosarcoma specimens^14^.

Histological characterization of lungs with metastases, obtained from the tibia injected mice, revealed the presence of a varying number of 143B tumor nodules in the lungs (example shown in Fig. 1d). Again, tdTomato IHC made it possible to clearly discriminate tumor cells from host cells. The tumor cells of these metastases were also found to have a strong expression of MT1-MMP (Fig. 1d).

In conclusion, our mouse model of osteosarcoma mimics the human disease, both with respect to the osteogenic and osteolytic properties and the expression of MT1-MMP in primary tumor cells. Importantly, MT1-MMP expression was retained in lung metastases.

### Generation and in vitro characterization of MT1-MMP deficient 143B cells

**Figure 2:**
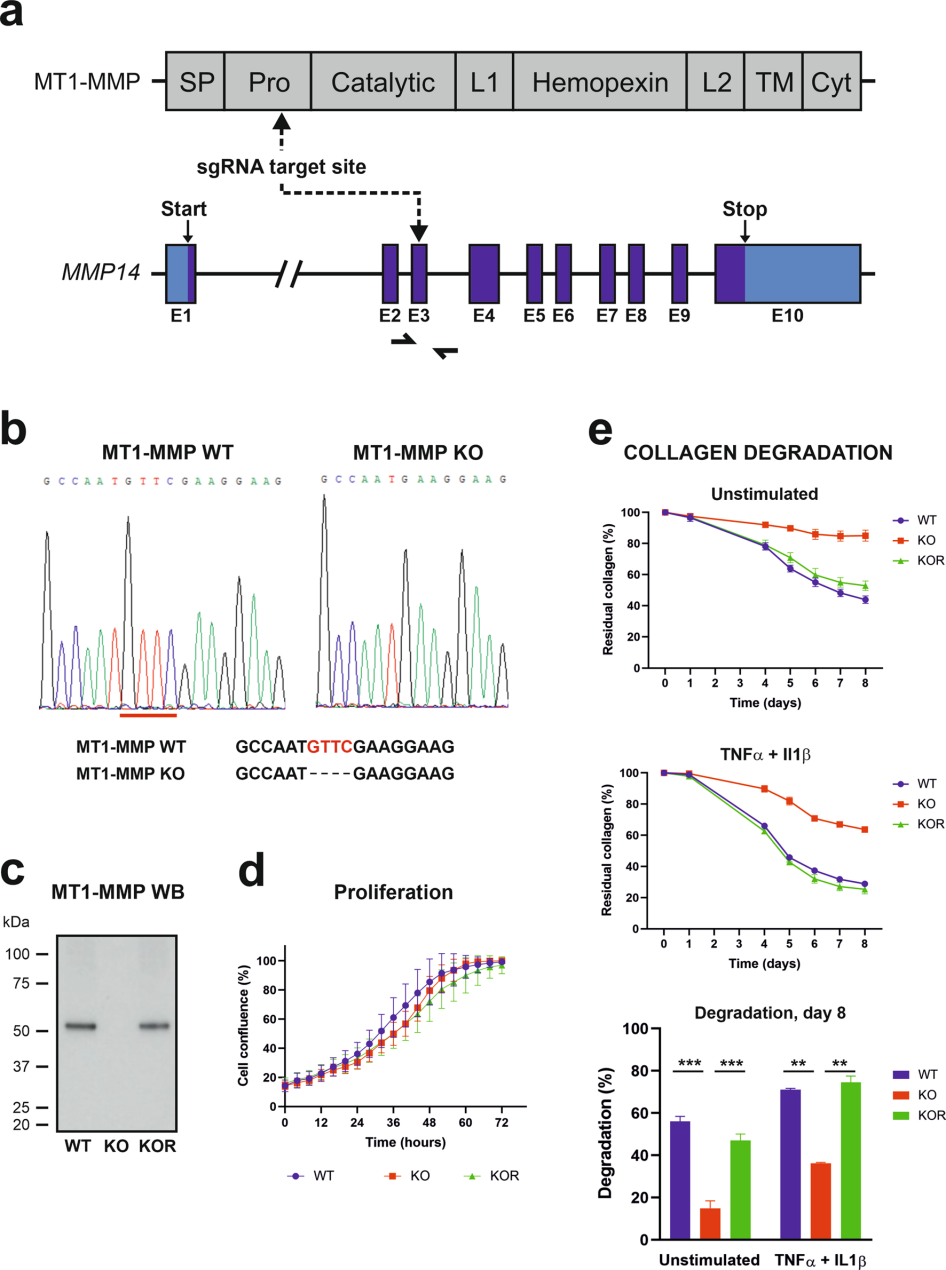
Generation and characterization of MT1-MMP deficient 143B cells. (**a**) Schematic overview of the CRISPR-manipulation strategy. The upper part depicts the domain composition of MT1-MMP protein and the lower part shows the organization of the human MT1-MMP gene (*MMP14*) with 10 exons (E1–E10). The sgRNA target site, as well as start and stop signals, are indicated. Half-arrows indicate the primers used for the generation of genomic PCR (gPCR) products for genotype determination. Protein domains: Signal peptide (SP), Pro-domain (Pro), Catalytic domain (Catalytic), Linker region-1 (L1), Hemopexin domain (Hemopexin), Linker region-2 (L2), Transmembrane domain (TM) and Cytoplasmic domain (Cyt). (**b**) DNA sequencing of the targeted region in CRISPR-manipulated cell lines. The genotype of monoclones was determined by DNA sequencing of gPCR products spanning the targeted region. Results from the selected 143B-luc2/tom WT and KO monoclones are shown. The KO clone contains a 4 nucleotide deletion (GTTC) and these 4 bases are underlined in the WT chromatogram and highlighted in the WT sequence. (**c**) western blot analysis of MT1-MMP protein expression in CRISPR-manipulated cells. Whole cell lysates prepared from 143B-luc2/tom MT1-MMP WT, KO and KOR monoclonal cell lines were analyzed for the expression of MT1-MMP by western blotting. Note the complete absence of MT1-MMP protein in the KO clone and the reappearance of the protein in the KOR clone. The raw data, including the entire chemiluminescence film and a Coomassie-stained gel for loading control, are available in the Supplementary material. (**d**) Growth curves of CRISPR-manipulated cells in vitro. Proliferation of the monoclones analyzed in (**c**) during a 3 day time course was monitored in real-time using the IncuCyte S3 imaging system. Data are presented as mean ± SD (n = 4). (**e**) Collagen degradation by 143B-luc2/tom MT1-MMP WT, KO and KOR cells. Cells were seeded on fluorescence-labeled collagen I matrices in the absence (upper) or presence (middle) of TNFα and IL1β. Fluorescence of the remaining matrix at any time point was measured on whole wells, and the proportion of residual collagen relative to wells without cells was calculated. The lower panel shows the proportion of degradation at day 8 (100%—proportion of residual collagen). Data are presented as mean ± SD (n = 2). One-way ANOVA was used to test for statistical significance; **p < 0.01, ***p < 0.001.

To enable studies on the role of tumor cell-expressed MT1-MMP in osteosarcoma progression, we generated MT1-MMP knock-out (KO) monoclonal cell lines, and corresponding MT1-MMP wildtype (WT) cell lines, by CRISPR/Cas9 gene editing (Fig. 2a–c). The editing strategy included transfection of the luciferase and tdTomato expressing 143B cell line with a plasmid encoding an sgRNA targeting a sequence in the N-terminal part of human MT1-MMP, as well as Cas9 and GFP^20^. Since the transfected cell population would include both MT1-MMP KO cells, resulting from frame shifts in the sequence encoding the pro-domain (Fig. 2a), and WT cells, resulting from successful repair after DNA cleavage or just unsuccessful cleavage, this procedure allowed the isolation of KO and WT clones that were maximally equivalent regarding pre-treatment. Consequently, the transfection was followed by single cell sorting of GFP positive cells to obtain monoclonal cell lines, from which we identified several MT1-MMP KO and WT clones. Among these, we selected single CRISPR-transfected WT and KO clones to be used in all subsequent in vitro and in vivo experiments investigating the role of MT1-MMP.

All clones were initially screened for MT1-MMP protein expression by western blot analysis and the genotype of potential WT and KO clones was subsequently verified by DNA sequencing of the targeted region. Results from the selected WT and KO clones, which both exhibited homogeneous DNA sequences, are shown in Fig. 2b,c.

To ensure that any functional alteration of the KO cells was linked to loss of MT1-MMP, we reintroduced the expression of the protease into these cells. This was done by subjecting the selected MT1-MMP KO cell line to precision CRISPR/Cas9-mediated homology-directed repair (HDR) using a single stranded DNA oligo donor as WT-template (see “Methods”). A monoclonal revertant cell line (MT1-MMP KOR) was isolated and the reappearance of MT1-MMP protein expression was confirmed by western blot analysis (Fig. 2c).

To address the function of MT1-MMP in vitro, we first analyzed the WT, KO and KOR clones using an IncuCyte S3 instrument to record and quantify their cellular growth in real-time. Reassuringly, as seen from the growth curves in Fig. 2d, all three cell lines exhibited very similar growth rates.

We then studied the capability of the CRISPR-manipulated cells to degrade collagen type I in vitro. Cells were seeded on fluorescence-labeled collagen type I matrices and collagen degradation was measured as a decrease in the matrix-associated fluorescence. A clear difference was observed between MT1-MMP-expressing and -deficient cells (Fig. 2e), with the WT and KOR cell lines degrading the matrix much more efficiently than the KO cells. Under these conditions, a threefold difference in degradation was observed at day 8. Stimulation with TNFα and IL1β increased the collagenolytic capacity of WT, KO and KOR clones, but the difference between the genotypes persisted under these conditions (twofold difference in collagen degradation at day 8).

Thus, the lack of MT1-MMP expression led to a strong reduction in cellular collagen-degrading capacity, although in this assay (Fig. 2e), a small residual activity was still present in the KO cells.

### MT1-MMP has no effect on osteosarcoma primary tumor development

**Figure 3:**
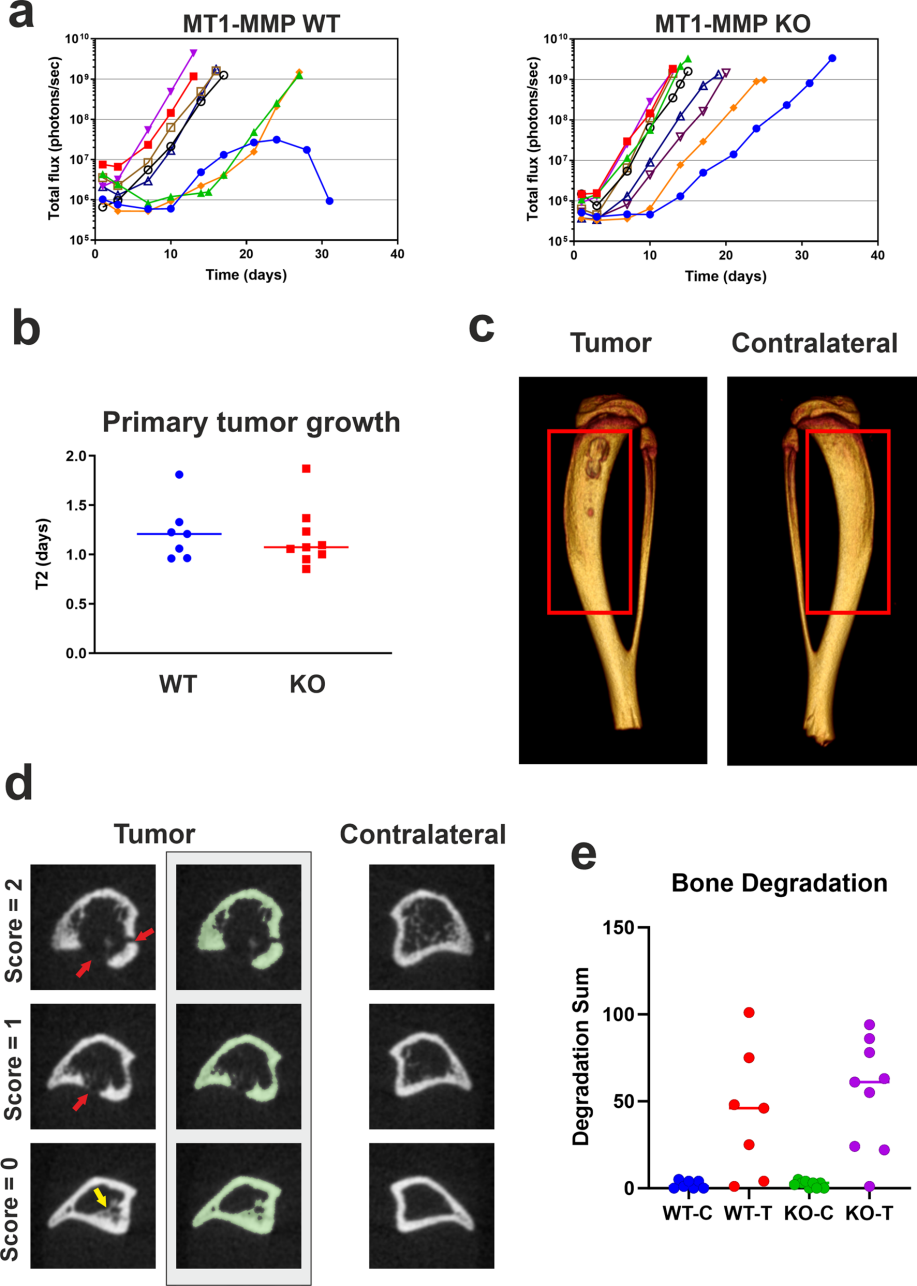
MT1-MMP is dispensable for 143B primary tumor growth and tumor induced bone degradation. (**a**) Primary tumor growth curves. Mice were injected with 3.3 × 10^4^ 143B-luc2/tom MT1-MMP WT or KO cells into the left tibia and primary tumor growth was measured twice weekly by bioluminescent imaging in vivo. Mice without tumor take were excluded. After an establishment phase of variable length, an exponential growth phase was observed in all mice except for one, where tumor growth ceased for unknown reasons (left, blue curve; excluded from subsequent studies). (**b**) Comparison of primary tumor doubling time (T2) within the exponential growth phase in mice with MT1-MMP WT and KO tumors. Median values are indicated. T-test revealed no difference between the genotypes (p = 0.7201). (**c**) µCT scanning of tumor tibiae. 3D reconstruction of a representative tumor tibia and corresponding contralateral control tibia. Red boxes indicate ROIs used for quantification of bone mineral density, bone volume and bone degradation. The current ROI is 7 mm long and comprises 235 2D µCT scanning images. (**d**) Bone degradation, new bone formation and scoring of degradation sites. µCT 2D images from different locations within the ROIs of the tibiae. The examples show tumor tibia images with areas of both bone degradation (red arrows) and new bone formation (yellow arrow). The equivalent locations in the contralateral tibia are shown. For each tumor bone image, the bone structure is shown (left column) along with the threshold-defined mask (pixel value 1500–6000, highlighted in green; right column). Visual examination revealed degradation and new bone formation in all included tumor bones irrespective of tumor genotype, except for one KO tumor. For quantitative analysis of bone degradation, each 2D image within the ROI of the tumor or contralateral bone was given a bone degradation score based on the number of holes extending through the cortex (red arrows; see also “Methods”). (**e**) Bone degradation results obtained for WT and KO tumors. For each tibia, all 2D images in the tibia ROI were examined individually and each image was given a score as defined in (**d**). The bone degradation sum was then calculated as the sum of all of the individual image scores. C: Contralateral bone; T: Tumor bone. Median values are indicated. T-test revealed no difference between the values obtained with the two tumor genotypes (p = 0.5331).

To evaluate the role of tumor derived MT1-MMP in local osteosarcoma progression, we compared the primary tumor growth in mice inoculated intra-tibially with 143B MT1-MMP WT or KO cells. A summary of the tumor characteristics is found in Table 1, while bioluminescence images of the mice at 24 h after injection are shown in Supplementary Fig. S1. The individual tumor growth curves are shown in Fig. 3a. The tumor take in both groups was equivalent to that observed in the pilot experiments (WT: 8/10; KO: 9/10). The growth patterns for the tumors of WT and KO origin were also similar. In both groups, an initial phase of establishment was followed by exponential growth (Fig. 3a). The length of the establishment phase varied considerably (3–10 days), but this variation in onset was observed in both groups. Once the tumors reached the exponential growth phase, they grew consistently with a mean doubling time of 1.2 days, with no difference between WT and KO tumors (Table 1 and Fig. 3b). Furthermore, there was no difference in the mean number of days to reach the experimental end point, which was defined by primary tumor size (Table 1). Based on these results, we conclude that the loss of MT1-MMP does not affect 143B orthotopic tumor growth. Mice injected with the KOR clone were included in the same experiment and the resulting tumors had growth characteristics similar to the WT and KO tumors, although for unknown reasons this clone displayed a low tumor take (6/10). Results obtained with the KOR clone are shown in Supplementary Fig. S2.

**Table 1:** Comparison of primary tumor characteristics following intra-tibial injection of MT1-MMP WT or KO 143B-luc2/tom cells.

	WT	KO	p-value
Primary tumor take	8/10	9/10	
Mean doubling time (days)^a^	1.22 ± 0.29	1.17 ± 0.30	0.7201
Mean time to reach experimental end point (days)^a^	18.1 ± 6.4	18.2 ± 6.6	0.9811
Mean IVIS signal at termination (photons/s × 10^9^)^a^^,b^	1.8 ± 1.1	1.9 ± 0.85	0.9616

^a^Includes all mice with a primary tumor, except one mouse where the primary tumor regressed after initial growth. Data are shown as mean ± SD.

^b^Time to reach experimental end point: day when total flux exceeded 10^9^ photons/sec.

### Loss of MT1-MMP has no effect on osteosarcoma-mediated bone destruction

Since MT1-MMP is a major collagenolytic protease in the bone compartment^2^, and since interference with collagen degradation may counteract osteosarcoma induced bone destruction^14^, it appeared likely that tumor-derived MT1-MMP would prove important in the processes of bone destruction associated with osteosarcoma growth and invasion. We therefore examined bone degradation in the tumor-inoculated tibiae by microcomputed tomography (µCT) scanning.

Consistent with the 143B tumor characteristics observed by histology (Fig. 1c), µCT scanning revealed areas of both bone degradation and new bone formation (Fig. 3c,d). We next sought to quantify and compare the amount of bone degradation induced by the WT and KO osteosarcoma cells. To this end, the simultaneous production of new bone matrix presented a challenge to obtain a quantification of degraded bone alone. Indeed, when using traditional measurements of bone degradation, such as bone mineral density (BMD) and bone volume (BV), many tumor-bearing tibiae had a higher BMD and BV than the non-tumor bearing contralateral tibia, despite extensive bone degradation in the tumor-bearing tibia (Supplementary Fig. S3). Therefore, we developed a quantification method based on measuring the number and size of degradation sites extending through the tibial cortex (Fig. 3d,e) and used this method to quantify and compare the amount of bone degradation in the tumor-bearing bones (Fig. 3e). To our surprise, no difference in the extent of bone degradation was found between MT1-MMP-expressing and MT1-MMP-deficient tumor cells. These results clearly show that tumor cell derived MT1-MMP is not required for bone degradation in the 143B model.

### Histological characterization of MT1-MMP WT and KO primary tumors

**Figure 4:**
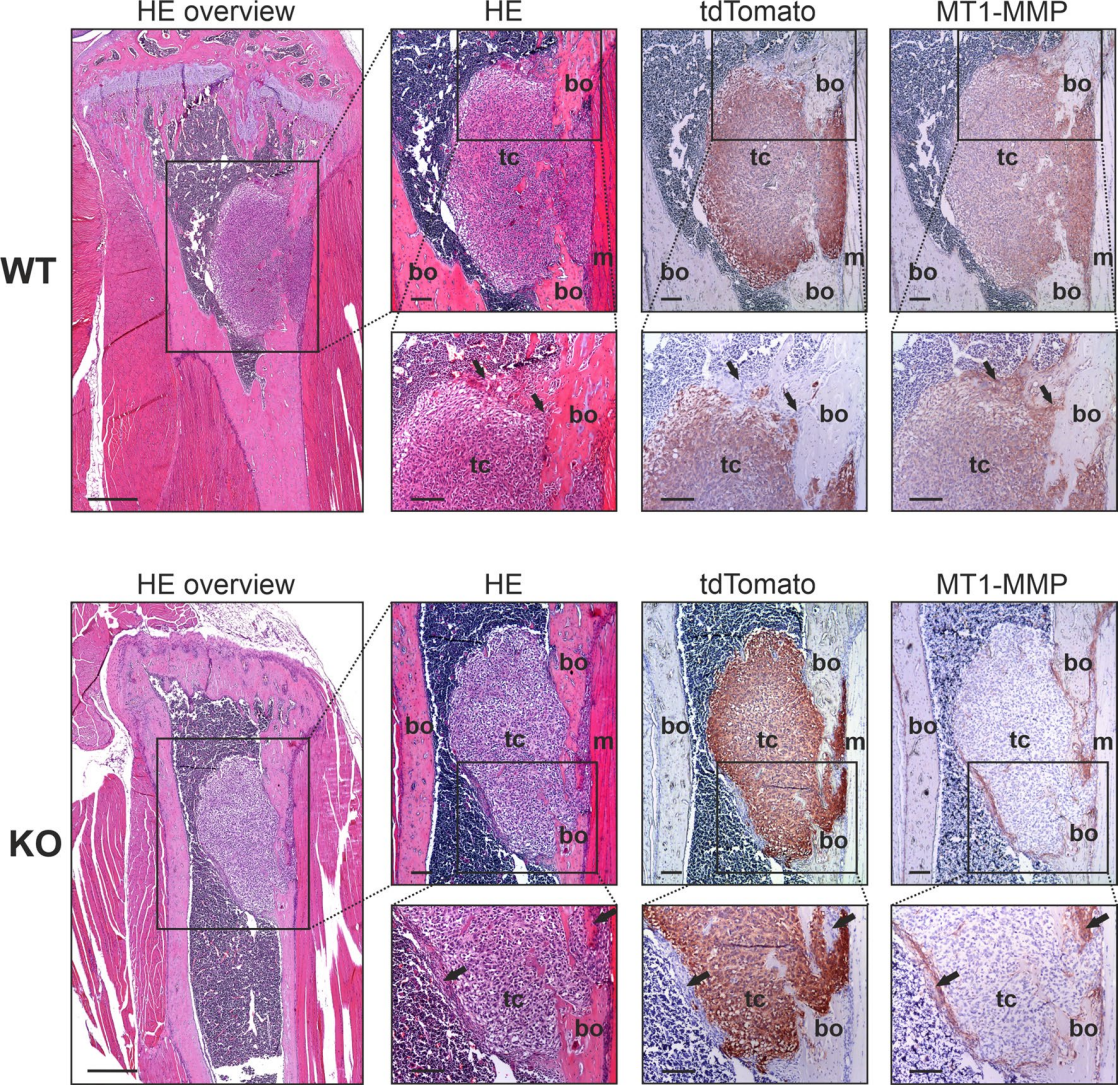
Histological characterization of primary tumors reveals no phenotypic difference between tumors of WT and KO origin**.** Representative examples of primary tumors originating from 143B-luc2/tom MT1-MMP WT and KO cells. Sections of the primary tumors were stained with HE or immunostained for tdTomato and MT1-MMP, respectively. Left: low magnification overview showing the upper part of the tibia, where the tumor is located (bars = 500 µm). Right: primary tumor close-ups in two different magnifications (bars = 100 µm). For each tumor, the box in the upper row indicates the region depicted in the lower row. Examples of tumor induced stromal responses are indicated with arrows; bo = bone; tc = tumor cells; m = muscle cells.

The absence of a role of MT1-MMP in primary tumor growth and bone degradation does not rule out that tumor cell derived MT1-MMP could have other roles in governing the tumor phenotype or microenvironment within the bone. Therefore, we performed a histological characterization of primary tumors derived from MT1-MMP WT and KO cells (Fig. 4). However, we did not observe any histological difference between tumors of WT and KO origin. In both groups, tumors were situated in the upper part of the tibia, the tumors grew invasively and, in accordance with the µCT scans, tumor growth was accompanied by both bone degradation and new bone formation.

In many cases, we found that tumor growth induced a stromal response within the bone. Thus, a layer of host cells was found in close contact with the tumor cells and the bone and these cells were clearly identified as non-tumor cells based on the absence of tdTomato expression (Fig. 4, arrows on lower panels). This stromal response was often found in areas of bone remodeling, but in some cases a layer of host cells between the tumor and the bone marrow was also observed. Examples of both types of host cell response are shown in Fig. 4. The primary tumor cells of WT origin displayed the expected expression of MT1-MMP, but the protease was also expressed on cells involved in the host cell responses. These MT1-MMP positive host cells were found associated with tumors of both WT and KO origin (Fig. 4). A morphological evaluation suggested that at least some of these cells were osteoclasts, which was supported by staining for TRAP activity (Supplementary Fig. S4).

Thus, although these observations did not exclude a role of stromally derived MT1-MMP (see “Discussion”), the lack of the protease on tumor cells did not influence the gross histological appearance, the overall balance between bone formation and degradation or the occurrence of a stromal response to tumor growth.

### MT1-MMP is not involved in the formation and growth of 143B lung metastases

Since MT1-MMP is crucial for the metastasis of mammary carcinoma and melanoma^7,10,21^, we also wanted to study the role of MT1-MMP in osteosarcoma metastasis. In the experiment shown in Fig. 3, lung metastases were indeed observed but in the majority of cases, these could only be detected after excision of the lungs and bioluminescence detection ex vivo. These lung metastases were found in mice injected with tumor cells of all of the three genotypes (WT, KO and KOR), although not in all mice (Supplementary Table S1). However, a closer examination of bioluminescence images, obtained in vivo throughout the study, revealed that the origin of these metastases was uncertain. Thus, in some cases we could detect 143B cells in the lungs less than 24 hs after intra-tibial tumor cell injection (examples shown in Supplementary Fig. S5). This suggests that, at least in our system, tumor cells may escape to the vasculature during the injection procedure, and that the observed lung tumor foci did not exclusively represent spontaneous metastases originating from the primary tumor. Since this observation severely questioned the use of our system as a spontaneous metastasis model, we decided to focus exclusively on the later stages in the metastatic process, including lung colonization and growth of lung tumor foci.

**Figure 5:**
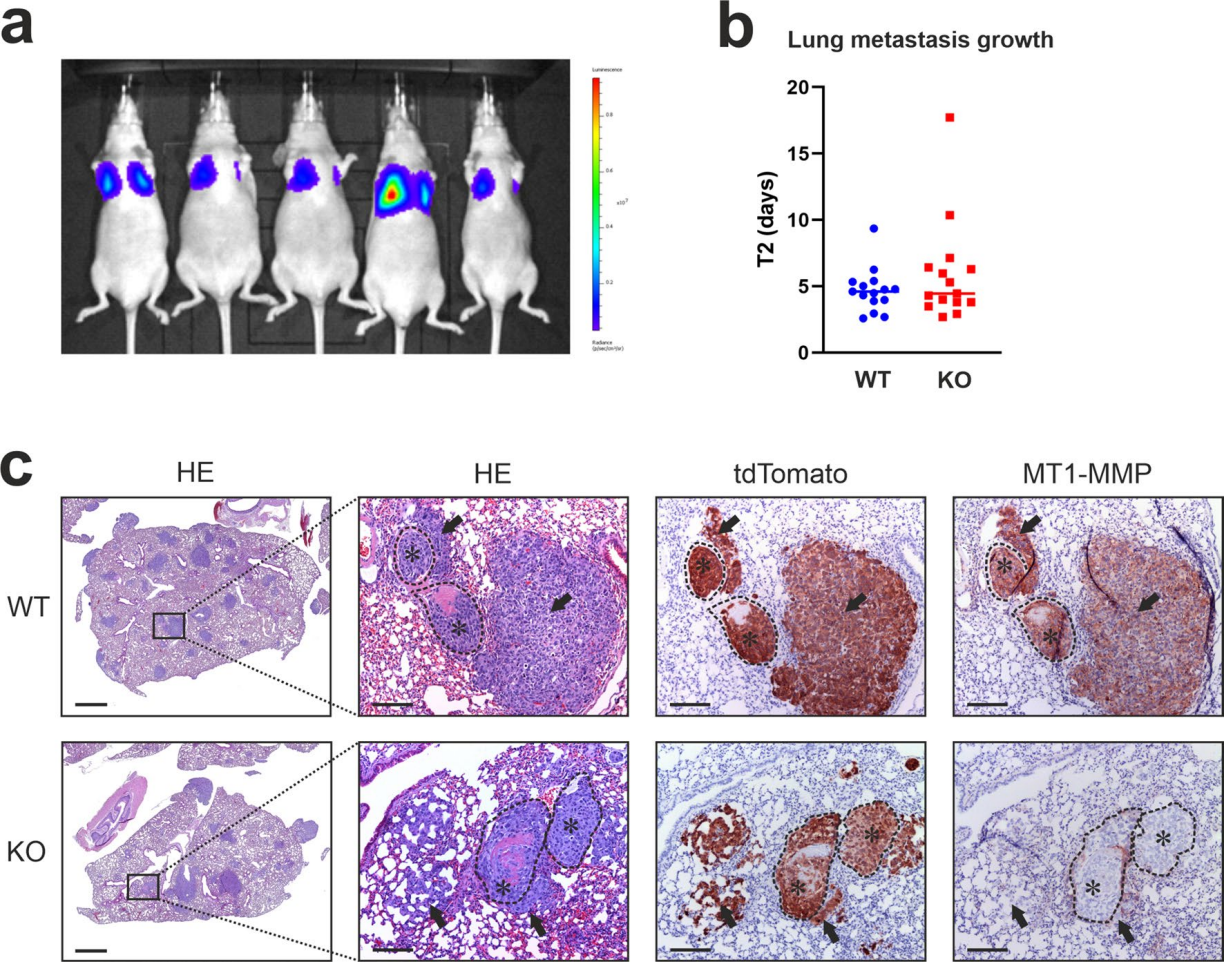
MT1-MMP is not required for the formation and growth of 143B metastases. (**a**) Bioluminescent detection of 143B-luc2/tom cells in the mouse lungs following i.v. tail vein injection. Examples of lung signals 20 hs after tumor cell injection are shown. (**b**) Comparison of metastasis doubling time (T2) from mice with MT1-MMP WT and KO lung metastases. Median values are indicated. T-test revealed no difference between the genotypes (p = 0.2676). (**c**) Histological characterization of lung metastases derived from MT1-MMP WT and KO cells. Representative examples of 143B metastases from both genotypes. Lung sections were stained with HE or immunostained for tdTomato and MT1-MMP, respectively. Left: low magnification images showing a lung lobe with 143B metastasis nodules (bars = 1 mm). Right: high magnification images of lung metastases (bars = 100 µm). The dotted lines indicate the presence of blood vessels. Examples of tumor cells within blood vessels (asterisks) and outside blood vessels (arrows) are shown for both genotypes.

Consequently, we turned to the 143B experimental metastasis model, where lung metastases form upon tail vein injection of tumor cells^17,22^. Mice were injected in the tail vein with 143B MT1-MMP WT or KO cells and the formation and growth of lung metastases were monitored weekly by bioluminescent imaging. Both groups displayed a 100% metastasis rate (Table 2) and in all mice, tumor cells were detected in the lungs on day one after injection (examples shown in Fig. 5a). The growth rate of the lung metastases was substantially lower than that of the primary tumors in the tibia (mean doubling time of 5.3 days and 1.2 days for the metastases and the primary tumors, respectively (Table 2 and Supplementary Fig. S6). However, when comparing the growth rates of MT1-MMP WT and MT1-MMP KO lung metastases, no difference was observed (Table 2 and Fig. 5b). Therefore, we conclude that MT1-MMP does not play a decisive role in the initial 143B lung colonization process or in the subsequent growth of 143B lung metastases.

**Table 2:** Comparison of lung metastasis characteristics following i.v. injection of MT1-MMP WT or KO 143B-luc2/tom cells.

	WT	KO	p-value
Lung metastases take	15/15	15/15	
Mean doubling time (days)^a^	4.7 ± 1.65	5.92 ± 3.82	0.2676

^a^Includes all mice. Data are shown as mean ± SD.

Since the extravasation process in particular might be dependent on proteases such as MT1-MMP, we also performed a histological evaluation of the 143B growth pattern in lung metastases arising from MT1-MMP WT and KO cells. However, we did not observe any histological difference between metastases arising from the two genotypes: In both groups, 143B metastases were spread across the pulmonary lobes and cells of both genotypes were situated both within blood vessels and in the lung parenchyma (examples shown in Fig. 5c). This demonstrates that tumor cell derived MT1-MMP is not essential for the capability of 143B cells to cross the vascular walls and settle down within the lung tissue.

## Discussion

MT1-MMP is a collagenolytic protease with a well-established and important role in collagen turnover in vivo, both during normal physiological bone development^2,23^ and during cancer invasion and metastasis (Reviewed by Turunen et al.^11^). In this study, the role of MT1-MMP during osteosarcoma progression was investigated and, intriguingly, we found that tumor cell expressed MT1-MMP is dispensable for both primary tumor growth, bone degradation and lung metastasis development in our model system of this disease. Although the biological variation in the tumor model makes it impossible to rule out that the protease could still play a minor role in some of these processes, our studies clearly showed that it is not an essential component on the tumor cells.

These findings were unexpected in the light of the large number of studies that have shown an active role of MT1-MMP in different types of cancer. Thus, as detailed below, this protease has been reported to be involved in several stages of cancer progression, including primary tumor growth, angiogenesis, tumor invasion and metastasis^7–10,21^. To our knowledge, the functional role of MT1-MMP in osteosarcoma has not been experimentally addressed, but expression studies clearly suggest an involvement in the progression of this disease. We have previously demonstrated the expression of MT1-MMP on the tumor cells in human osteosarcoma specimens using IHC^14^ and in a transcriptome analysis of human osteosarcoma samples, MT1-MMP was found to be among the 10 most upregulated genes^24^. Furthermore, high MT1-MMP expression levels are correlated with poor survival in osteosarcoma patients^25,26^.

With the findings of the current study being unexpected, it is necessary to consider if any methodological limitations might have influenced the result. We believe that within the limits of biological variation, the sum of our approaches have allowed a rigorous quantitative comparison of the studied tumor genotypes. Several features of our experimental set-up contribute to this notion. As a model system for osteosarcoma, we first optimized the widely used 143B mouse osteosarcoma model. A common limitation when measuring tumor growth in orthotopic osteosarcoma models is the lack of detection of tumors until they have degraded the cortical bone and invaded into the surrounding muscle tissue. Thus, many studies only measure primary tumor growth after tumor escape from entrapment within the bone and development into large, palpable tumor masses^16,17,27^. To circumvent this problem, some authors have used osteosarcoma cells transfected with luciferase to follow tumor growth^28–31^. We employed the same strategy, supplemented with tdTomato transfection to facilitate histological detection of tumor cells. The double-transfected 143B cell line made it possible to measure primary tumor growth non-invasively within the bone by bioluminescent imaging and to collect the bones at a tumor progression stage where the processes of bone turnover were still active and could be studied by histology. This procedure also made it possible to collect tumor bones with equivalent primary tumor sizes for subsequent analysis by µCT and histology.

Furthermore, we developed an optimized injection procedure, which allowed us to accurately inject a low number of tumor cells in a small volume. With these methodological improvements, the primary tumors never became macroscopically visible in our experiments and by subsequent histological analysis, we could study the processes of bone degradation and new bone formation in the tibia. Other advantages of the bioluminescent approach included early detection of lung metastases and comparison of growth rates between tumors at different anatomical sites. Finally, the histological characterization demonstrated that MT1-MMP is expressed by the tumor cells both during primary tumor growth and in pulmonary metastases, suggesting that the 143B model is indeed a suitable system to study the role of MT1-MMP in osteosarcoma development.

As a central precondition for our in vivo study, the genetic manipulation successfully affected the matrix-degrading capacity of the osteosarcoma cells. Thus, knock-out of the MT1-MMP gene led to 143B cells with a strongly reduced collagen degrading activity in vitro (Fig. 2e). This reduction could be reverted by reintroducing MT1-MMP expression in the KOR clone, providing an important rescue control since the current study was based on a comparison of single monoclonal cell lines. In the in vivo experiments, the rescue clone turned out to be less important since MT1-MMP gene knock-out in its own right had no effect on the tumor characteristics. However, as would be expected, tumors formed by the KOR clone displayed growth and bone degradation similar to the WT and KO clones (Supplementary Fig. S2).

The pronounced effect of MT1-MMP gene knock-out observed in vitro makes it particularly striking that none of the functions studied in vivo were affected. Whereas we found the intra-tibial growth of osteosarcoma cells to be independent of MT1-MMP, several studies have reported that blocking of MT1-MMP reduce in vivo growth of human cancer cell lines of various origins^8,21,32^.

Likewise, the lack of effect of MT1-MMP deficiency in bone degradation was unexpected in the light of the successful targeting of the cell’s ECM degrading capacity. We have previously shown that blocking the endocytic collagen receptor uPARAP decreases osteosarcoma induced bone destruction^14^. This cell-surface associated receptor binds extracellular collagen fragments, produced by limited extracellular cleavage, and routes them for lysosomal degradation^33^. At least in vitro, a coupling has been demonstrated between the uPARAP-dependent endocytic process and the initial MT1-MMP-mediated collagen cleavage^34^. Furthermore, whereas uPARAP deficiency in mice only leads to a relatively mild bone phenotype^35,36^, MT1-MMP deficient mice display a severe phenotype in the bone compartment^2^. This phenotype is exacerbated in mice double-deficient for MT1-MMP and uPARAP^35^, demonstrating a role of both of these components in collagen turnover processes during bone growth. However, in the case of osteosarcoma-directed bone degradation, the role of MT1-MMP appears less crucial.

This also applies for the balance between bone deposition and turnover in the current disease model. µCT analysis revealed areas of both extensive bone destruction and new bone formation and around half of the tumor bones had a higher BMD and BV than the contralateral control bone. This phenomenon was observed irrespective of the genotype of the inoculated tumor cells (Supplementary Fig. S3). A similar phenomenon was observed by Labrinidis and co-workers, who reported a 36% increase in bone volume of osteosarcoma cell injected tibiae compared with the corresponding non-tumor bearing tibiae^37^. Importantly, these authors did not observe this phenomenon with osteolytic breast cancer and myeloma cell lines. Therefore, also in transplanted mouse models, the increased bone deposition is an osteosarcoma-specific feature.

To enable quantification of bone degradation, we utilized a quantification method based on the number and extension of degradation sites that extended through the tibial cortex. However, the lack of effect of MT1-MMP manipulation was evident irrespective of the quantification method used (BMD, BV or bone degradation) (Fig. 3e and Supplementary Fig. S3). In particular, the µCT analysis demonstrated extensive bone degradation in tumor-bearing bones from both WT and KO injected mice and histological analyses confirmed the presence of bone degradation areas, as well as the presence of tumor cells outside the bone, in tumor-bearing bones from both groups (Fig. 4).

Methodological considerations were also necessary in our studies on osteosarcoma metastasis. The origin of lung metastases in orthotopic osteosarcoma mouse models is debated: two studies suggest that they are spontaneous metastases derived from the primary tumor only^16,38^, one study points towards the metastases being derived from both the primary tumor and from direct seeding to the lungs in connection with the injection procedure^39^ and, finally, one recent study indicates that all pulmonary metastases are the results of immediate tumor cell seeding^40^. In all of these studies, osteosarcoma cells were injected into the tibia and the origin of the metastases was evaluated by subsequent removal of the primary tumor by amputation of the tumor-bearing limb. Our setup with luciferase-transfected cells allowed the detection of tumor cells with a high sensitivity, and in some cases we observed bioluminescent lung signals less than 24 hs after tumor cell injection. This observation indicates that, in these cases, the pulmonary metastases were derived from immediate seeding, in line with the findings of Maloney et al.^40^. Importantly, the bioluminescent lung signal was much lower than the primary tumor signal, and it was only detected because we utilized a wide range of imaging settings. This opens the possibility that undetected tumor cell micro foci may also have been present at an early stage in the lungs of mice examined in some of the previous studies. It remains unknown whether the conflicting results regarding the origin of pulmonary metastases are caused by the use of different osteosarcoma cell lines, differences in the number and volume of injected cells, differences in detection methods or other methodological differences.

Although this limitation excluded a controlled investigation of spontaneous metastasis in our mouse model, the experimental metastasis model based on i.v. tumor cell injection allowed us to study the later stages of the metastastic process and the role of MT1-MMP therein. These stages include extravasation, invasion into the metastatic site and local growth at this site. The lack of importance of 143B MT1-MMP expression in these processes was unexpected. For example, in similar studies, blocking of MT1-MMP has been reported to reduce the number of experimental pulmonary metastases in mice with melanoma cells injected i.v.^21^. Furthermore, in a recent study it was shown that knock-out of the gene encoding tetraspanin-28 (CD81) in 143B cells led to a pronounced reduction in the extravasation process occurring during experimental lung metastasis, thus inducing tumor cell arrest in the vessels^41^. It was hypothesized that this effect was related to a reduced expression of various MMPs in the CD81 KO cells, including MT1-MMP. While this is clearly a possibility, knock-out of the MT1-MMP gene alone did not have a similar effect.

Whereas the absence of MT1-MMP in osteosarcoma cells did not lead to any appreciable effect on tumor growth, bone degradation or experimental metastasis, it is not known why the role of the protease is less pronounced in these tumors than that reported in other tumor models. Either MT1-MMP activity is completely dispensable for the processes investigated in this study, or the loss of MT1-MMP activity on the tumor cells is compensated by other proteases or by MT1-MMP expressed by non-malignant cells in the tumor microenvironment. In the light of the collagenolytic activity of MT1-MMP, it is noteworthy that another important collagenase, MMP-1, has been shown to be involved in osteosarcoma growth and metastasis in mouse models^42^. Other relevant proteases in this respect might include membrane-associated MMPs other than MT1-MMP^11^.

Regarding the possibility of a proteolytic contribution from the stromal cells, we did observe a tumor-induced host cell response in the primary tumors and MT1-MMP was indeed expressed by these host cells, some of which appeared to be osteoclasts. This response was found in tumors of both MT1-MMP WT and KO origin (Fig. 4 and Supplementary Fig. S4). Since osteoclasts are involved in tumor-induced bone destruction, it cannot be excluded that an osteoclast contribution of MT1-MMP activity could be involved in that event. Thus, a recent report has revealed a function of osteoclast MT1-MMP in non-malignant bone resorption^43^, opening the possibility that a similar process could be in play in our system. However, also in the non-malignant process, MT1-MMP was dispensable, since a redundant bone-directed activity of another protease, MMP-9, was found in the same study^43^. Consequently, MMP-9 is yet another protease that could be involved in the osteosarcoma-induced processes.

The expression of MT1-MMP by other host cells might also provide some compensation for the lack of tumor cell expressed MT1-MMP in the KO tumors. In this connection, it is noteworthy that expression studies have shown that MT1-MMP is expressed by both tumor cells and tumor-associated stromal cells in human cancers of various origins^44^. However, using a murine genetic breast cancer model, Feinberg et al. recently showed that whereas deletion of stromally derived MT1-MMP had no effect on tumor progression, deletion of carcinoma cell MT1-MMP decreased both local cancer invasion and metastasis^10^. Our mouse model with orthotopic osteosarcoma cells clearly reveals a different pattern.

In conclusion, we have shown that the pronounced role of MT1-MMP in cancer invasion and metastasis is not universal. This emphasizes the need for detailed studies on the tumor and stromal expression of this protease in several different cancers, as well as its functional role in well-characterized tumor models with a high degree of resemblance with human disease.

## Methods

### Cells

143B cells were purchased from ATCC and cultured in MEM GlutaMAX with 1 × MEM non-essential amino acids, 10% fetal bovine serum (FBS) and 1% penicillin–streptomycin.

### Generation of a 143B cell line with stable expression of luciferase and tdTomato

143B cells were stably transfected with the vector pFULT expressing firefly luciferase 2 and tdTomato^45^ using Lipofectamine 3000. To ensure high expression of luciferase and tdTomato, Zeocin treated cells were subjected to single cell sorting on a FACS-Aria (BD Bioscience), using high tdTomato fluorescence as selection criterion. The monoclone, 143B-luc2/tom-1A6, was selected for CRISPR-manipulation.

### CRISPR/Cas9

MT1-MMP-deficient 143B monoclonal cell lines were generated using CRISPR/Cas9 following published protocols^20^. All oligonucleotides used are listed in Supplementary Table S2. Briefly, a sgRNA targeting MT1-MMP exon 3 was designed using an online tool (http://cripsr.mit.edu)^20^. sgRNA oligonucleotides were cloned into CRISPR plasmid PX458 (Addgene), which also encodes Cas9 and GFP. The 143B-luc2/tom-1A6 cell line was transfected with PX458/MT1-MMP sgRNA using lipofectamine 3000. Cells with medium GFP expression were single cell sorted. Selected clones were subjected to western blot analysis and DNA-sequencing, and one WT (WT-3C11) and one KO (KO-1E9) clone were chosen for in vivo experiments.

To reintroduce expression of MT1-MMP in MT1-MMP KO cells, the sequence of the KO-1E9 clone, which harbors a 4 nt deletion (GTTC), was reverted back to WT sequence by CRISPR/Cas9-mediated HDR, thereby creating a KO-revertant (KOR). This was achieved using a ssODN as template (Supplementary Table S2) and following the CRISPR/Cas9 strategy described above. To this end, a KOR sgRNA was designed to target the KO-1E9 mutation and cut at a position + 1 residue relative to the ”GTTC” deletion/insertion site. Revertant monoclones were identified by RFLP analysis of digested PCR amplicons (see Supplementary Table S2). Selected clones, which exhibited the expected RFLP and contained a correct homogeneous MT1-MMP WT DNA sequence, were further validated by western blotting.

To obtain DNA sequences of clones*,* genomic DNA was extracted using QuickExtract DNA extraction solution (Cambio). CRISPR-manipulated regions were amplified by gPCR, amplicons purified using QIAquick PCR purification kit (Qiagen), and sequenced by Sanger sequencing.

### Western blot

Cell lysates were prepared by lysing EDTA-detached cells for 20 min at 0 °C in lysis buffer (1% Triton X-100, 10 mM Tris/HCl, 140 mM NaCl, pH 7.4) with 0.5% protease inhibitor cocktail III (Calbiochem). 25 µg cell lysate was separated by SDS-PAGE on 4–12% gradient gels under non-reducing conditions, followed by electroblotting onto PVDF membranes. Blocking, washing, incubation with antibodies and chemiluminescent substrate were performed using the Anti-Rabbit Western Breeze Chemiluminescent Immunodetection Kit (Thermo Fisher). Rabbit anti-MT1-MMP mAb (Abcam Ab51074/Clone EP1264Y) was used at 0.25 µg/ml. Samples to be compared were run on the same gel and detection of chemiluminescence was performed using uniform exposure of the PVDF membrane.

### Cell proliferation assay

Cellular growth was monitored in real-time using IncuCyte S3 imaging system (Essen Bioscience). Briefly, cells were seeded at 5000 cells/well in a 96-well plate and within the instrument, microscopic live-cell phase contrast images were captured every 4 h for 72 h using a 10× objective lens (4 images per well). Cell confluence (based on area metrics) was analyzed and quantified using the integrated cell confluence algorithm. Similar results were obtained in 3 independent experiments.

### In vitro collagen degradation

The collagen degrading capacity of selected cell lines was measured using reconstituted native, rat tail type I collagen (VWR) matrices prepared from a mixture of non-labeled collagen (Col1) and fluorescently labeled collagen (Col1-A647)^46^. In brief, wells in 24-well plates were coated with 300 µl 0.3 mg/ml Col1 in 20 mM acetic acid overnight at 37 °C. Next, wells were washed with water and filled with 100 µl gels, made from a neutralized mixture of Col1 (0.38 mg/ml) and Col1-A647 (0.02 mg/ml). Gels polymerized for 2 h at 37 °C followed by drying for two days at RT. For experiments, rehydrated gels were washed in PBS, and cells seeded at 100,000 cells/well in medium with or without addition of 10 nM TNFα and 325 pM IL1β (Peprotech). Collagen gel-associated fluorescence of whole wells was measured on a Licor Odyssey (LI-COR Biosciences) before and after seeding cells, and daily afterwards. Wells without cells and wells without fluorescence were included as internal controls. For each well, residual collagen was calculated as percentage of fluorescence of wells without cells (i.e. without degradation) and normalized to day 0. Degradation was calculated as 100 – residual collagen (%).

### Animal experiments

All in vivo tumor experiments were performed with female nude mice (NMRI-Foxn1^nu/nu^ mice from Janvier Labs).

For orthotopic primary tumor growth and bone degradation, cells were injected into the left tibia of 5 weeks old mice under isoflurane anaesthesia. Cells were harvested with Trypsin/EDTA, and resuspended in MEM GlutaMAX. Pilot experiments using injection of 1 × 10^4^, 3.3 × 10^4^ or 1 × 10^5^ cells in 8–10 µl medium indicated that a tumor take of > 80% could be obtained with 3.3 × 10^4^ cells. In the experiment comparing MT1-MMP WT and KO cells, 3.3 × 10^4^ cells in 8 µl were injected. One h prior to injection, mice were given s.c. injections of 5 mg/kg Carprofen and 0.1 mg/kg Buprenorphine. Intra-tibial injection has been described previously^47^, but was further optimized. Thus, gently drilling was first performed with a 26G Atraucan needle (Ø 0.47 × 25 mm; BRAUN) to gain access to the proximal tibia through the tibial plateau. Then, the stylet was removed and, using a 100 µl Hamilton syringe equipped with a 30G removable needle, 8 µl cell suspension was slowly injected through the Atraucan needle. Mice received a daily s.c. injection of 5 mg/kg Carprofen for a period of three days following the injection for pain management. Primary tumors were detected and quantified by bioluminescent in vivo imaging one day after cell injection and subsequently twice a week until the tumors reached the study endpoint (termination criterion: primary tumor total flux > 10^9^ photons/s).

For experimental metastasis, cells were harvested with Trypsin/EDTA and resuspended in PBS + 3% FBS to a final concentration of 1 × 10^7^ cells/ml. Cell suspensions of 200 µl were injected into the right lateral tail vein of 9 weeks old mice using a 27G needle. Lung metastases were detected and quantified by bioluminescent in vivo imaging one day after tail vein injection and subsequently once a week until they reached the study endpoints (lung metastasis total flux > 10^9^ photons/sec or t = 11 weeks).

### Bioluminescent in vivo imaging

Bioluminescent in vivo imaging was performed using In Vivo Imaging System (IVIS) Lumina II (Perkin Elmer) and the integrated isoflurane based anaesthesia unit. Anaesthetized mice were injected s.c. with 150 mg D-Luciferin/kg body weight (Perkin Elmer) and monitored continuously in the IVIS 15–30 min post-injection. Peak signal intensity was quantified using Living Image Software. In all experiments, a standard size ROI was used to quantify either the primary tumor signal or the lung metastasis signal.

### Collection of tissue

When a primary tumor or metastasis signal reached study endpoints, a final in vivo imaging was performed. Mice were then euthanized and relevant tissues were collected for histology. All tissues were incubated in 4% PFA for 24 h (lungs) or 72 h (bones), followed by transfer to 70% ethanol for long-term storage.

### µCT scanning

For determination of bone degradation, tumor tibiae and contra-lateral control tibiae underwent scanning by µCT using an Inveon Multimodality PET/CT scanner (Siemens). µCT images were acquired using 360 projections, 60 kV, 500 mA, 1300 ms exposure and reconstructed (Feldkamp algorithm) with an isotropic voxel size of 0.032 mm. Image files were exported as DICOM files and quantitative analysis was performed using OsiriX open-source image software.

### Quantification of bone degradation

Evaluation of µCT results and quantification of bone degradation were performed on blinded µCT data. For each tumor tibia and contra-lateral control tibia, quantitative analyses were performed on the DICOM exported 2D image stack of cross-sectional scanning pictures. To ensure that an equivalent part of the tibiae was used for quantification and compensate for variation in bone length between mice, a region of interest (ROI) was defined based on two anatomical reference points. The lower anatomical reference point was defined as the point where the tibia and fibula merge. The upper anatomical reference point was defined as the proximal tip of the fibula. The ROI was then defined as 80% of the images between the two anatomical reference points, extending from 10 images below the upper reference point and downwards. Importantly, ROI only contained tibia bone structures, ROI length was identical for any given tumor bone and corresponding control bone, and, in all tumor tibiae, ROI included all areas of bone degradation. Due to variation in bone length, ROI size varied between mice, but on average a ROI was 7.8 mm long and contained 245 2D images (maximum number 270; minimum number 224). For quantification of various measures of bone degradation, all bone structures within ROI was first selected as being between pixel threshold values 1500–6000 (64 bit). BMD was calculated as the cumulative pixel value of all selected bone structures and BV was calculated as the volume of selected bone structures. For each mouse, relative tumor bone integrity was calculated as BMD_tumor_/BMD_control_ or BV_tumor_/BV_control_ ratio. For quantification of degradation sites extending through the tibial cortex, all 2D images in the tumor tibia ROI were examined individually and each image was given a score based on number of cortical holes in the image. Score 0 = 0 holes; score 1 = 1 hole; score 2 = 2 holes etc. (examples shown in Fig. 3d). For each tumor tibia, a degradation sum was calculated as the sum of individual image scores.

### Processing of mouse tissue

Bones were decalcified in 10% EDTA pH 7.4 (EDTA-sol), by microwave heating at 50 °C, 600 W for 20 min followed by 50 °C, 300 W for 2 h, and transferred to fresh EDTA-sol for 3 days at 4 °C. Finally, they were rinsed under running de-ionized water for 1 h. Lungs and decalcified bones were dehydrated with ethanol, followed by Tissue-Tek Tissue Clear (Sakura), and embedded in paraffin. Tissues were sectioned at 3.5 µm on a microtome.

### Immunohistochemistry

Tissue sections were deparaffinized and rehydrated using Tissue-Tek Tissue Clear and decreasing concentrations of ethanol, followed by rinsing in water. Sections for IHC were pretreated with 5 µg/ml proteinase K (Roche) in 50 mM Tris, 0.5 M EDTA, pH 8 at 37 °C for 15 min, followed by blocking of endogenous peroxidase activity with 1% H_2_O_2_ for 15 min. Immunostainings were performed using rabbit anti-RFP pAb (2 µg/ml, ROCKLAND, # 600–401-379) and rabbit anti-MT1-MMP mAb (3.7 µg/ml, Abcam, ab51074, EP1264Y). Primary antibodies were diluted in Background Reducing Antibody Diluent (Agilent). Sections were incubated with primary antibodies overnight at 4 °C, washed in TBS-T (50 mM Tris, 150 mM NaCl, 0.5% Triton X-100, pH 7.6) and incubated 45 min with EnVision + System HRP Labelled Polymer Anti-Rabbit IgG (Agilent). Chromogen staining was performed using NovaRED HRP substrate kit (VWR). Nuclear counterstaining was done using Mayer’s haematoxylin (Histolab). After staining, sections were dehydrated with increasing concentrations of ethanol, followed by Tissue-Tek Tissue Clear and mounted using Tissue-Tek Tissue Mount (Sakura). TRAP staining was performed as described^14, 48^. Overview pictures were obtained with a Nanozoomer Digital Pathology slide scanner and close-up pictures were taken with a camera connected to a Leica DM2500 microscope.

### Statistics

A one-way ANOVA was used to test for statistical significance in the in vitro experiment shown in Fig. 2e. For experiments in vivo, the study was designed to demonstrate a hypothetical large difference between the studied groups of mice. With a 50% difference in the parameter under study and assuming a standard deviation of 25%, the use of 7 mice in each group would allow for a high power (95%) for demonstration of the difference with a P-value of < 0.05 (two groups of mice; two-tailed Student’s t-test). When comparing three groups using a two-sided One-way ANOVA test, a power of 90% would be obtained with the same number of mice per group and the same values for P and standard deviation. To compensate for the expected incomplete tumor take, we included 10 mice in each group, except for the study with experimental metastasis, where a particularly high biological variation was to be expected. In the latter experiment, 15 mice were used in each group. A two-tailed Student’s t-test was used to test for statistical significance in Figs. 3, 5, Tables 1 and 2. A P-value of < 0.05 was considered significant for all statistical tests.

### Approval for animal experiments

All mouse experiments were performed in accordance with license from the Danish Animal Experiments Inspectorate, license number 2014–15–0201–00322 (L. H. Engelholm). The project protocol was submitted to the Department of Experimental Medicine, the University of Copenhagen (formal authority for veterinarian examination and approval of project plans involving animals at the University animal facility). Approval of the protocol, reference no. P 16-398, was granted to L. H. Engelholm.

## Supplementary information

Supplementary Information.

## Data Availability

The transfected cells and datasets, generated during and/or analysed during the current study, are available from the corresponding author on reasonable request.
