# Supplementary material for: Tumor cell MT1-MMP is dispensable for osteosarcoma tumor growth, bone degradation and lung metastasis
Source: Sci Rep. 2020 Nov 5;10:19138. doi: 10.1038/s41598-020-75995-6 (PMC7645741; doi:10.1038/s41598-020-75995-6)
Supplement: Supplementary file 1 — Supplementary Information. [file 41598_2020_75995_MOESM1_ESM.pdf]

Supplementary information

Tumor cell MT1-MMP is dispensable for osteosarcoma tumor growth, bone degradation and lung metastasis

Signe Z. Ingvarsen[1], Henrik Gårdsvoll[1], Sander van Putten[1], Kirstine S. Nørregaard[1], Oliver Krigslund[1], Josephine A. Meilstrup[2], Collin Tran[3], Henrik J. Jürgensen[1], Maria C. Melander[4], Carsten H. Nielsen[5], Andreas Kjaer[5], Thomas H. Bugge[3], Lars H. Engelholm[1] & Niels Behrendt[1*]

[1]Finsen Laboratory, Rigshospitalet/Biotech Research and Innovation Center (BRIC), University of Copenhagen (UCPH), 2200 Copenhagen N, Denmark. [2]BRIC, UCPH, Copenhagen, Denmark. [3]Proteases and Tissue Remodeling Section, National Institute of Dental and Craniofacial Research, National Institutes of Health, Bethesda, MD, U.S.A. [4]In Vivo Pharmacology, Symphogen A/S, Ballerup, Denmark. [5]Department of Clinical Physiology, Nuclear Medicine & PET and Cluster for Molecular Imaging, Rigshospitalet and UCPH, Copenhagen, Denmark.

***Address all correspondence to:**

Niels Behrendt, Section head, D.Sc.

Finsen Laboratory, Rigshospitalet/BRIC, the University of Copenhagen, Ole Maaløes Vej 5, DK-2200 Copenhagen N, Denmark

Tel.: (+45) 3545 6030. E-mail: niels.behrendt@finsenlab.dk

Supplementary material includes Supplementary Figures S1-S6, Supplementary Tables S1 and S2 and Raw data for Article Fig. 2C (Western blot and loading control).

Supplementary Fig. S1

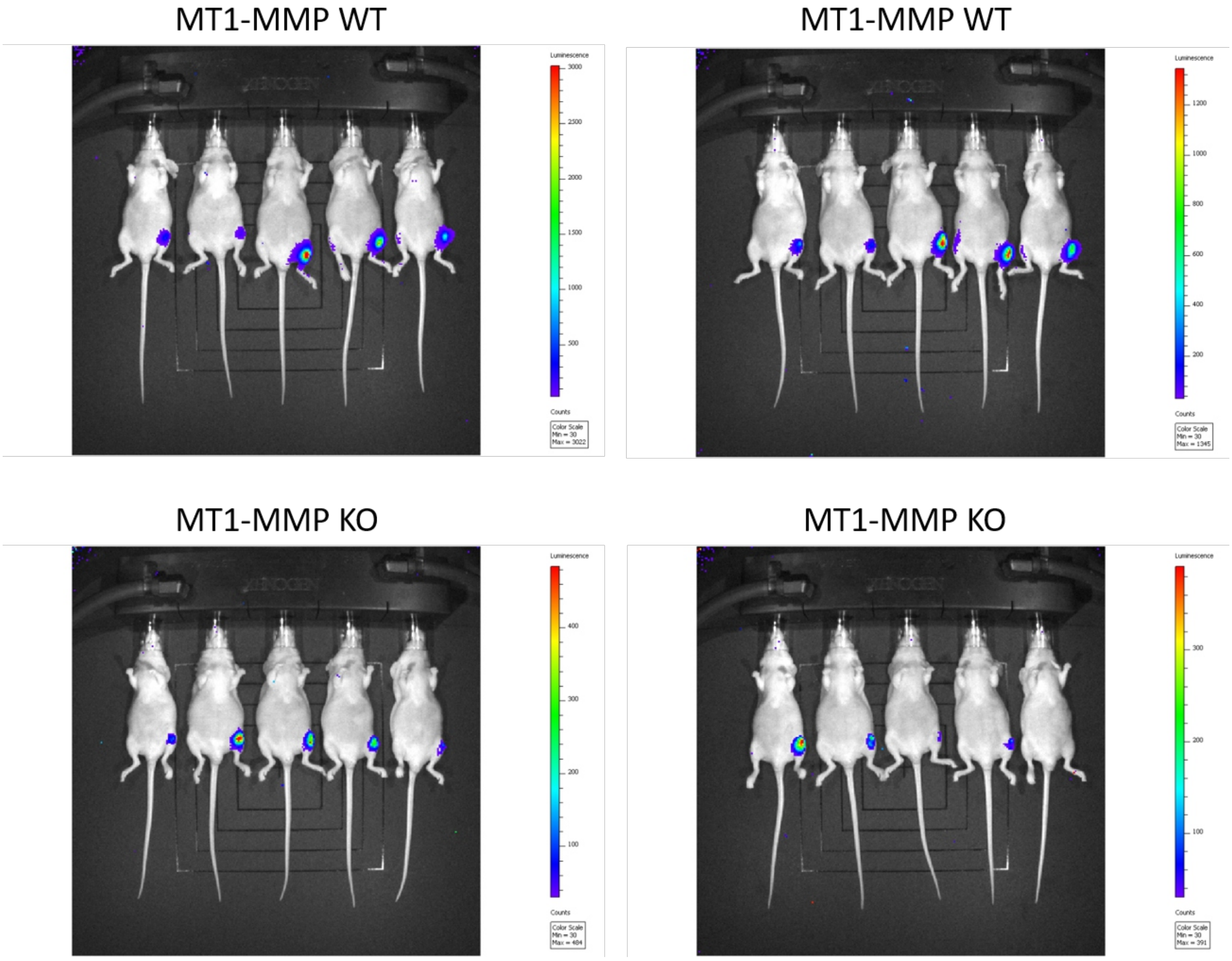

Bioluminescence images of mice at 24 hs after intra-tibial injection

Tumor cell genotypes (WT, KO) are indicated. Intratibial signals are observed in 10/10 and 9/10 mice for the WT and KO tumors, respectively.

Supplementary Fig. S2

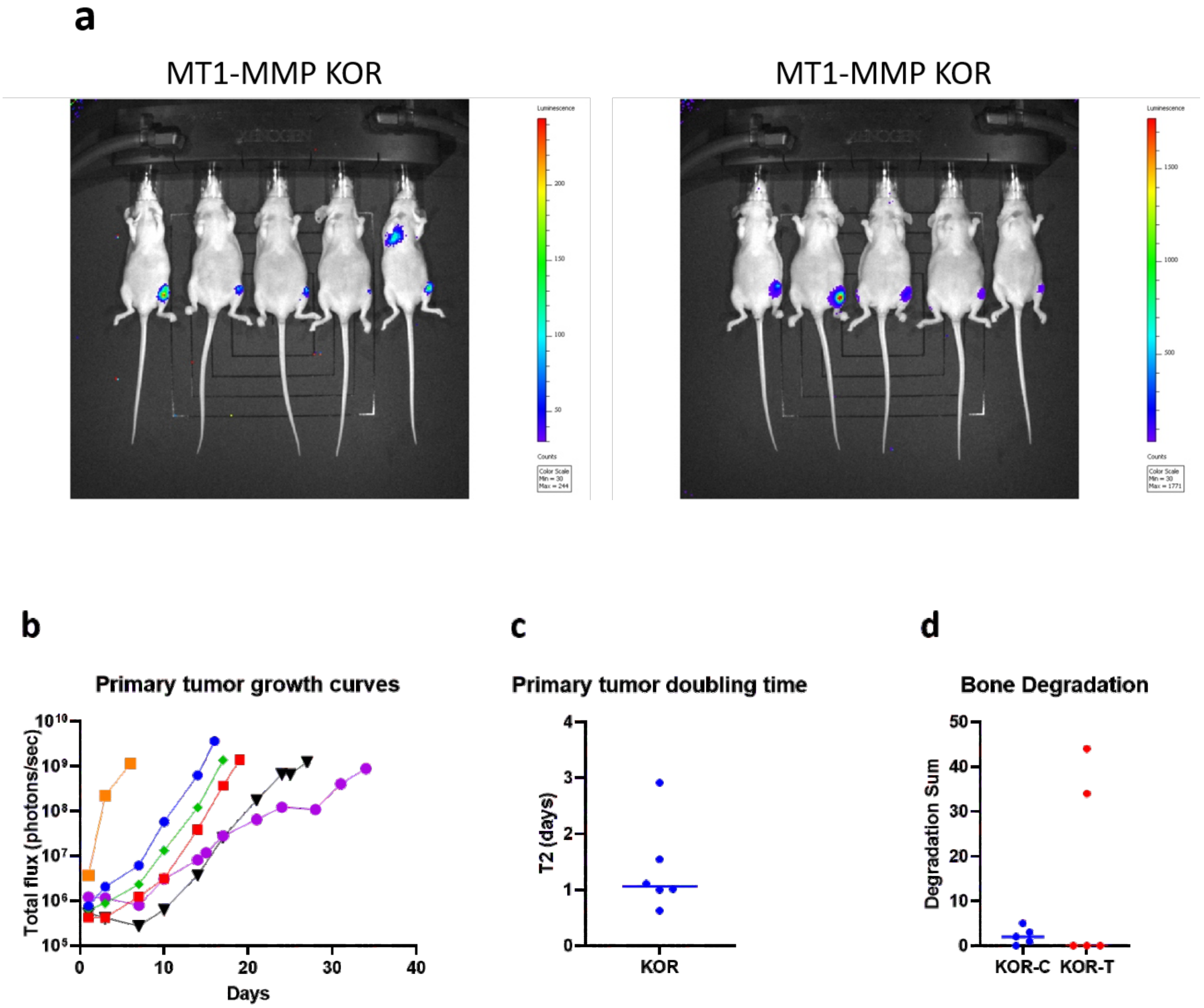

Tumor growth and bone degradation in mice with intra-tibial MT1-MMP knock-out revertant (KOR) tumors

a, Bioluminescence images of mice at 24 hs after intra-tibial injection.
b, Tumor growth curves. Mice without tumor take were excluded. A lag phase followed by exponential growth was observed in all mice except for one, where no lag phase was evident (light brown curve; excluded from subsequent studies).
c, Primary tumor doubling time (T2) within the exponential growth phase.
d, Bone degradation, using quantification method shown in Fig. 3d and e. Although three mice showed degradation sum 0 (lack of complete penetration of bone cortex), the µCT examination showed evidence of bone degradation in all tumor-bearing bones except for one.

Supplementary Fig. S3

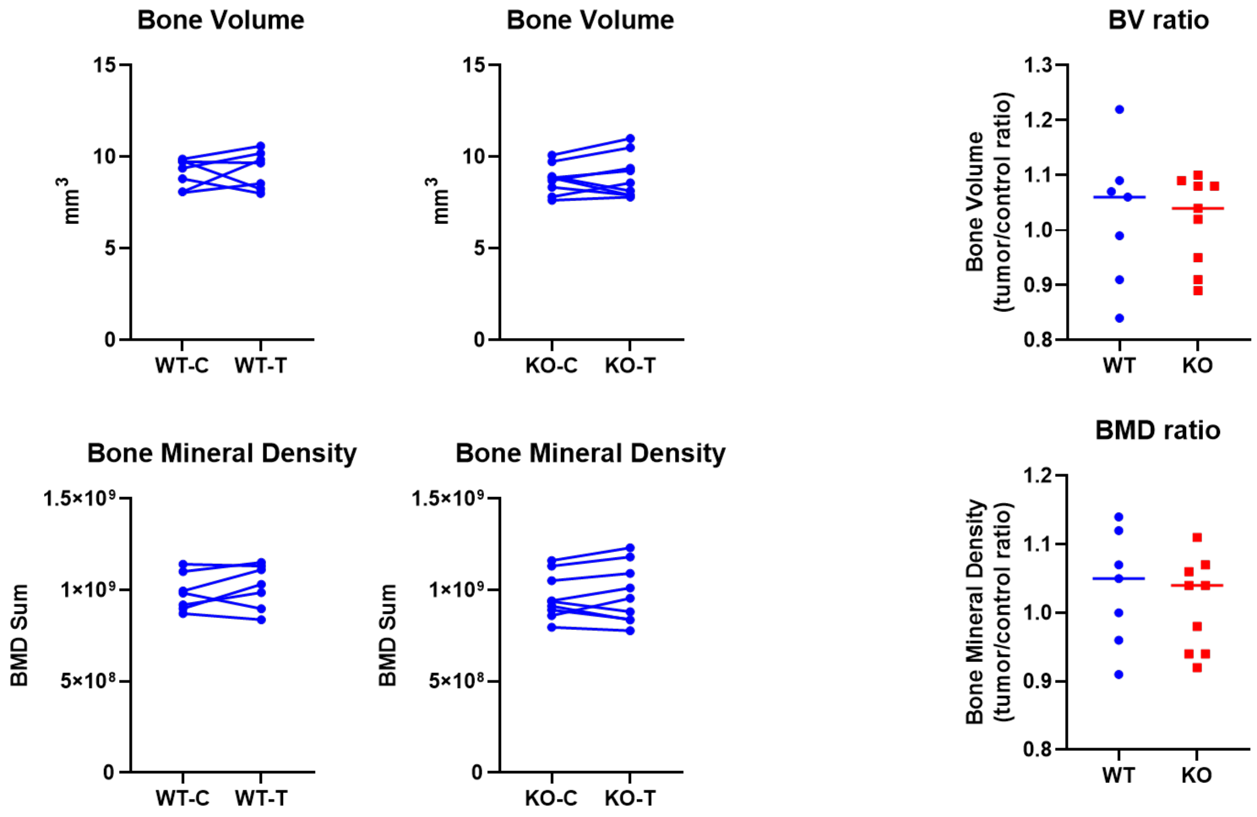

Bone volume and bone mineral density in tibia-injected mice

For all mice with a primary tumor (Fig. 3), the bone volume (BV) and bone mineral density (BMD) of the tumor and control (contralateral) tibia ROIs were calculated. For each mouse, the result is shown as a pairwise comparison of tumor and contralateral bone (left) and as the tumor/control ratio (right). C: Contralateral bone. T: Tumor bone. Median values are indicated in the right panels. T-test revealed no difference between tumor/control ratios of WT and KO tumors. BV ($p = 0.8800$); BMD ($p = 0.5259$).

Supplementary Fig. S4

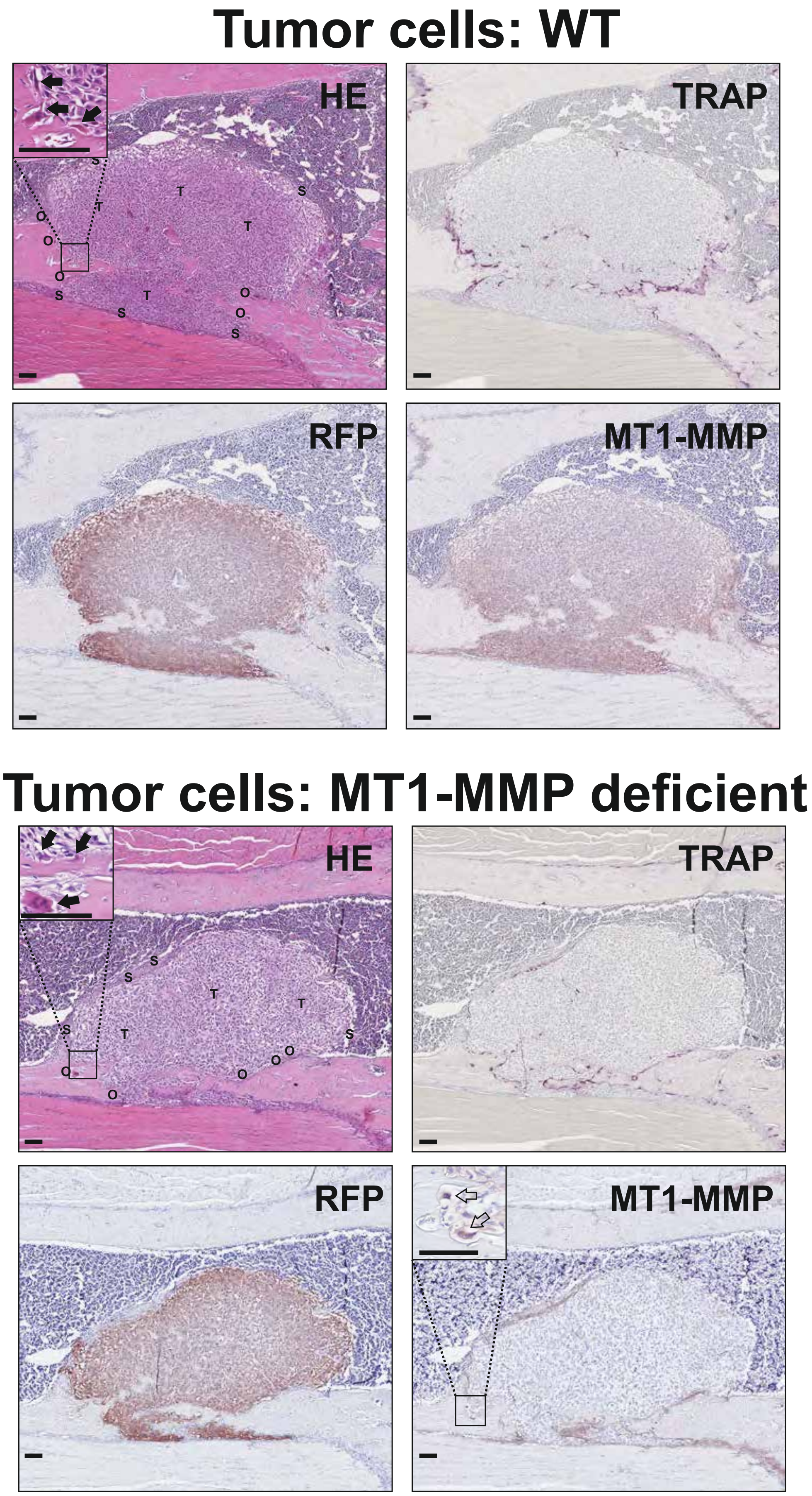

MT1-MMP-positive osteoclasts and stromal cells in 143B bone tumors

Sections of bones carrying 143B-luc2/tom MT1-MMP WT or KO tumors (MT1-MMP deficient) were stained with HE, immunostained for tdTomato and MT1-MMP, or stained for TRAP activity . Arrows in inserts with higher magnification indicate cells with osteoclast morphology, including some MT1-MMP positive cells. o = osteoclast; t = tumor cells; s = stromal cells. Bars = 100 µm

Supplementary Fig. S5

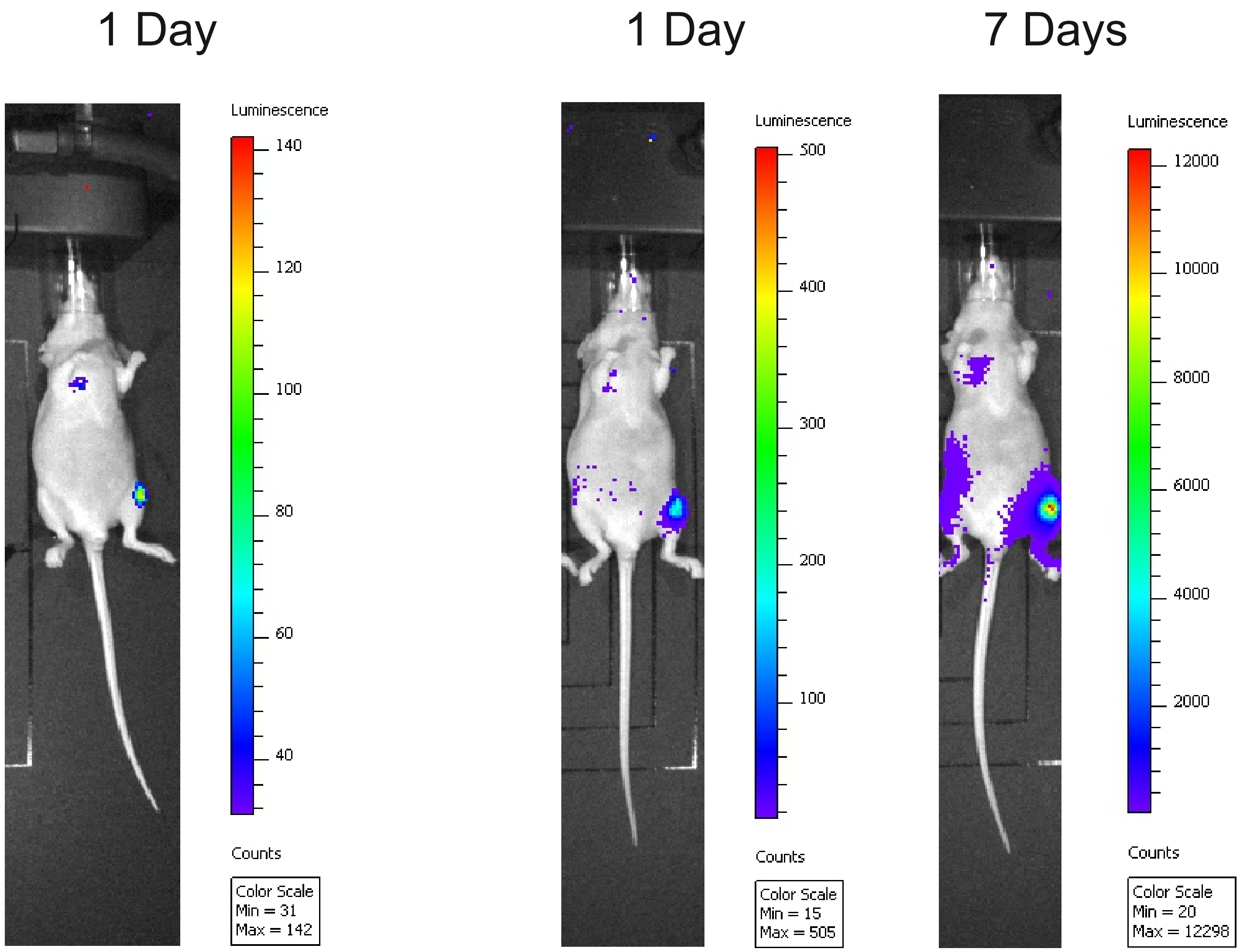

Pulmonary seeding of 143B cells following intra-tibial injection

Examples showing mice with lung foci resulting from immediate tumor cell seeding. Mice were injected with 143B-luc2/tom cells into the left tibia and monitored by bioluminescence imaging, either 1 day or 7 days after tumor cell injection. Left: Mouse from pilot investigation, injected with 1x10^5 cells, examined after 1 day. Right: Mouse from the experiment shown in Fig. 3 and Supplementary Fig. S1 (injection of 3.3x10^4 cells), examined 1 day and 7 days after injection, using high detection sensitivity. Lung metastases in this mouse were confirmed at termination by bioluminescence imaging *ex vivo* (Supplementary Table S1).

Supplementary Fig. S6

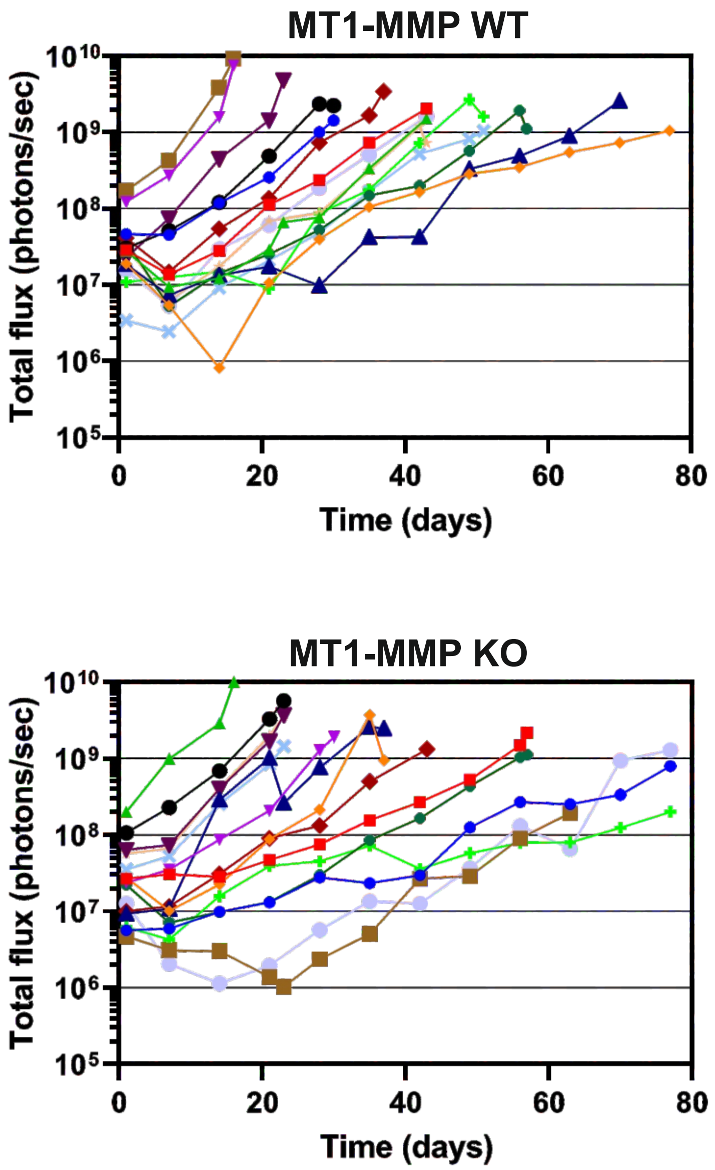

Lung metastasis growth curves following tail vein injection of 143B WT or MT1-MMP KO cells

Mice were injected with 2x10^6 143B-luc2/tom MT1-MMP WT or KO cells into the right lateral tail vein. Lung metastasis growth was measured weekly by bioluminescent imaging *in vivo* and represented in terms of the obtained bioluminescent signal (see Fig. 3a).

Supplementary Table S1: Lung metastases in tibia-injected mice[1]

Tumor genotype	Number of mice included[2]	Mice with quantifiable lung signal[3]	Mice with lung signals below quantification limit[3]	Mice with uncertain lung status	Mice with no lung signals observed
MT1-MMP WT	7	1	2	4	0
MT1-MMP KO	9	1	2	2	4
MT1-MMP KOR	5	1	1	1	2

[1] Lung metastases were detected by bioluminescence examination of excised mouse lungs at termination.

[2] Examination of lungs was performed for all mice in the experiment with intratibial injection of mice with WT, KO and KOR tumors, except for mice excluded due to lack of tumor take or abnormal tumor growth (Fig. 3 and Supplementary Fig. S2).

[3] A quantifiable signal was defined as Total flux > 5×10^5 photons/sec

Supplementary Table S2: DNA oligonucleotides used during CRISPR/Cas9 procedures[1]

Name	Sequence 5' to 3'
MT1-MMP sgRNA upper	CACCGTAGCGCTTCCTTCGAACAT
MT1-MMP sgRNA lower	AAACATGTTCGAAGGAAGCGCTAC
MT1-MMP genomic PCR Fw	CACCCCACTCCCCCATATCTC
MT1-MMP genomic PCR Rev	TATGTGGCATACTCGCCCACC
PX458 U6-Fw sequencing primer	GAGGGCCTATTTCCCATGATTCC
MT1-MMP sgRNA KOR 1E9 upp	CACCGGCGTAGCGCTTCCTTCAT
MT1-MMP sgRNA KOR 1E9 low	AAACATGAAGGAAGCGCTACGCC
MT1-MMP ssODN KOR 1E9 upp[2]	CCCCGATGTGGTGTTCCAGACAAGTTTGGGGCTGAGATCAAAGCCAAT**GTTC**GAAGGAAGCGCTACGCCATCCAGGGTCTCAAATGG

[1]All DNA oligonucleotides were delivered by LGC Biosearch Technologies, Risskov, Denmark.

[2]To perform HDR on the MT1-MMP KO-1E9 an 87 nt long ssODN template was designed to contain the missing "GTTC" nucleotides inserted centrally (labelled red in sequence). Furthermore, a BstBI restriction enzyme site (underlined in sequence) is destroyed in the KO-1E9 mutant, but restored in the KO-revertant (KOR). The oligo was modified by introduction of phosphorothioate bonds between the first 3 nucleotides at the 5'-end and the last 3 nucleotides at the 3'-end of the oligo to inhibit exonuclease degradation.

Raw data for Western blot and loading control (Article Fig. 2C)

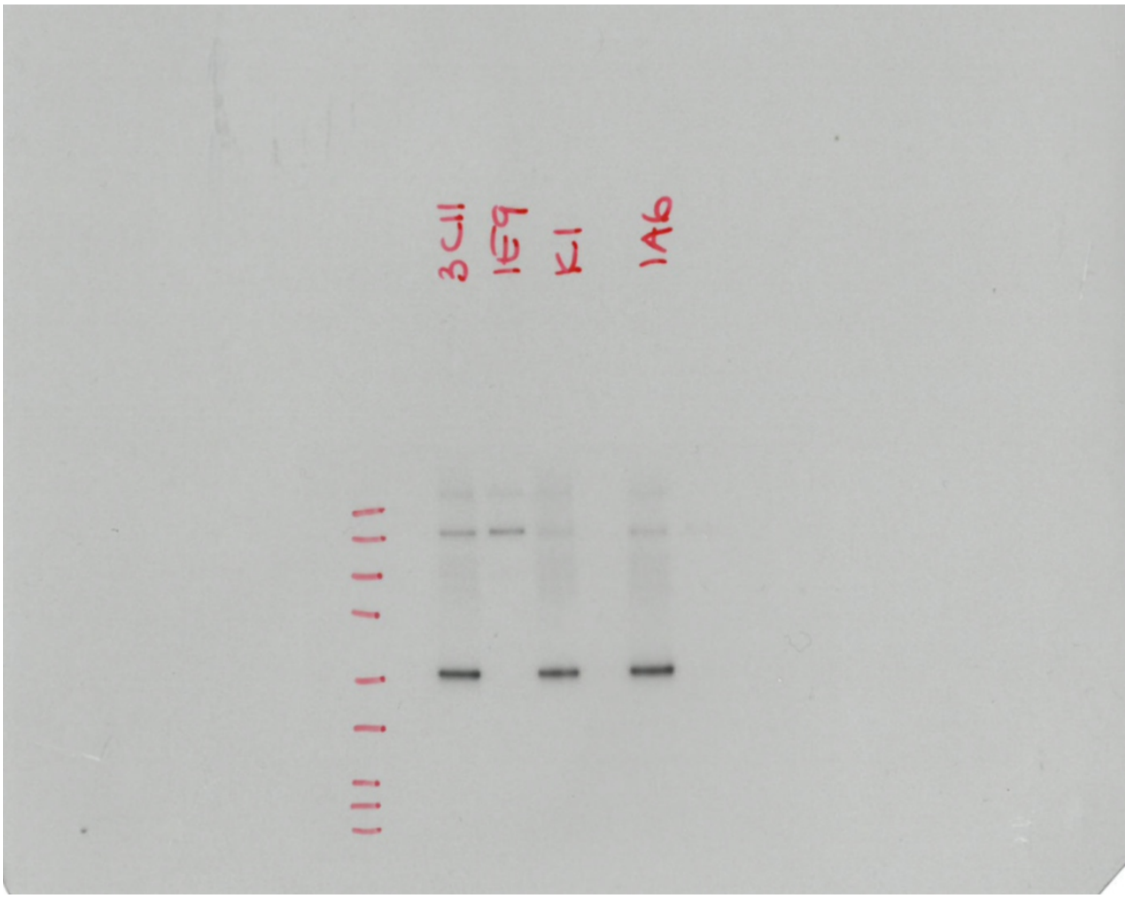

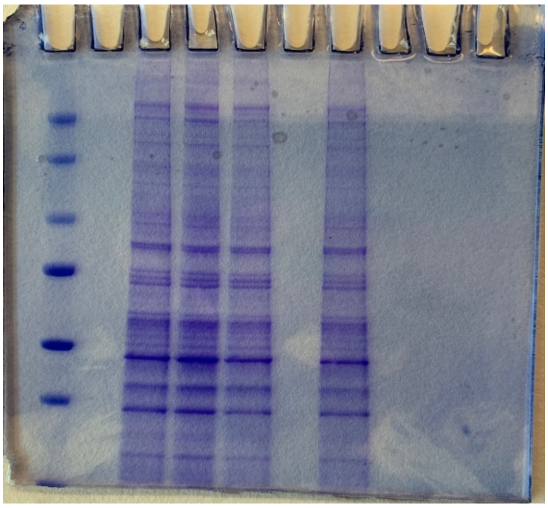

Top: Chemiluminescence film after exposure of Western blot PVDF membrane with samples from 143B-luc2/tom MT1-MMP WT, KO and KOR monoclonal cell lines, resolved and detected as described in the legend to Fig. 2C. 3C11: WT cells; 1E9: KO cells; K1: KOR cells; 1A6 (not included in article Fig. 2C): Irrelevant cells with positive expression of MT1-MMP. An empty lane separates sample K1 from sample 1A6. Red markings to the left of the lanes: Mr markers, from top to bottom (kDa): 250 – 150 – 100 – 75 – 50 – 37 – 25 – 20 – 15.
Bottom: Coomassie stained SDS gel serving as a loading control for the Western blot shown to the left.
